# Older Adults’ Loneliness, Social Isolation, and Physical Information and Communication Technology in the Era of Ambient Assisted Living: A Systematic Literature Review

**DOI:** 10.2196/28022

**Published:** 2021-12-30

**Authors:** Rita Latikka, Rosana Rubio-Hernández, Elena Simona Lohan, Juho Rantala, Fernando Nieto Fernández, Arto Laitinen, Atte Oksanen

**Affiliations:** 1 Faculty of Social Sciences Tampere University Tampere Finland; 2 Faculty of Built Environment Tampere University Tampere Finland; 3 Faculty of Information Technology and Communication Sciences Tampere University Tampere Finland

**Keywords:** loneliness, social isolation, older adults, physical information and communication technology, systematic literature review

## Abstract

**Background:**

Loneliness and social isolation can have severe effects on human health and well-being. Partial solutions to combat these circumstances in demographically aging societies have been sought from the field of information and communication technology (ICT).

**Objective:**

This systematic literature review investigates the research conducted on older adults’ loneliness and social isolation, and physical ICTs, namely robots, wearables, and smart homes, in the era of ambient assisted living (AAL). The aim is to gain insight into how technology can help overcome loneliness and social isolation other than by fostering social communication with people and what the main open-ended challenges according to the reviewed studies are.

**Methods:**

The data were collected from 7 bibliographic databases. A preliminary search resulted in 1271 entries that were screened based on predefined inclusion criteria. The characteristics of the selected studies were coded, and the results were summarized to answer our research questions.

**Results:**

The final data set consisted of 23 empirical studies. We found out that ICT solutions such as smart homes can help detect and predict loneliness and social isolation, and technologies such as robotic pets and some other social robots can help alleviate loneliness to some extent. The main open-ended challenges across studies relate to the need for more robust study samples and study designs. Further, the reviewed studies report technology- and topic-specific open-ended challenges.

**Conclusions:**

Technology can help assess older adults’ loneliness and social isolation, and alleviate loneliness without direct interaction with other people. The results are highly relevant in the COVID-19 era, where various social restrictions have been introduced all over the world, and the amount of research literature in this regard has increased recently.

## Introduction

Loneliness and social isolation can occur at any stage of human life. Particular attention has been paid to these circumstances among older adults, an increasing demographic in many societies. In 2050, over 20% of the population of most countries will be over 60 years old [1], and there is a strengthening global trend of those living alone later in their life [2]. During old age, the size of one’s social network and the extent of social activities are likely to reduce [3-5]. This topic has also become timely due to the COVID-19 crisis where various country-level restrictions and governmental recommendations on social distancing have been introduced [6,7]. These factors together bring forth the importance of addressing loneliness and social isolation among older adults.

Loneliness refers to perceived social isolation or a subjective unpleasant and distressing feeling that results from a significant discrepancy or mismatch between one’s actual and desired social relationships [8-10]. In the typology of social and emotional loneliness, social loneliness is characterized by the lack of engaging social networks, and emotional loneliness refers to the lack of close emotional attachment [11,12]. Social isolation typically concerns an objectively limited or a lack of social contact with others [8], and some of its common quantifiable markers are a shortage in one’s social contacts and network size [13]. Despite their similarities, loneliness and social isolation are not the same [12,14]. Loneliness is a subjective emotional feeling, whereas social isolation describes an objective and a quantifiable aspect of social relationships [13]. For instance, the quality of social relationships is more closely related to loneliness compared to the quantity of social relationships [12,15,16].

Recent prevalence estimates from the United States show that more than 40% of older adults are lonely (29% occasionally and 19% frequently) [8,17]. In Europe, prevalence estimates range from Central and Eastern Europe’s 30%-55% to Northwestern Europe’s 10%-20% [18,19]. As for social isolation, a recent estimate considers 24% of older adults aged 65 and above as socially isolated [20]. Various demographics can also be used for prevalence estimation purposes [21]. Prevalence estimates fluctuate across research studies due to differences in the considered populations, measures, age groups, sample sizes [22], definitions, intensity and duration of the experience [23], and cultural differences [24]. The extent to which people are willing to self-report their loneliness experiences needs to be critically considered. For example, research indicates that men are more reluctant to admit their loneliness than women, likely due to a stigma associated with it [25]. However, instead of asking about loneliness directly, indirect validated measures can also be applied [17].

Loneliness and social isolation are significant predictors of mortality [13,14,26,27], and they are associated with poorer physical and mental health [8]. For instance, loneliness is associated with poorer cardiovascular health [28], lower cognitive function [29,30], depression [31,32], anxiety, suicide ideation [33], higher psychological distress [34,35], lower self-esteem [36], sleep and stress problems [37,38], and health behaviors such as lower physical activity [39]. In turn, social isolation is associated with lower self-rated physical health [40], lower health-related quality of life and health status [41], worse cardiovascular and mental health [42], and increased vulnerability to dementia [43]. Therefore, it is evident that solutions to combat both circumstances are needed.

Partial solutions to assess loneliness and social isolation among older adults have been sought from the field of information and communication technology (ICT). Previous literature reviews have examined empirical studies on various types of technologies and their effectiveness in alleviating social isolation [44]. Other reviews address interventions targeting loneliness and social isolation among older people, which include technological and nontechnological approaches [45,46]. There are also reviews on diverse technologies and caregiving that have identified their impact on loneliness and social isolation alleviation, among other effects [47,48]. These studies address loneliness and social isolation from the perspective of fostering social networking and support, together with community interaction and engagement.

However, we assume that there are habits other than communication with other humans that can also be related to loneliness and social isolation, and these habits can be assessed and tracked using novel intelligent technologies. In particular, robots, wearables, and smart homes hold potential value in this area. In this review, these technologies are grouped under the term “physical ICT,” broadly referring to physical technologies able to collect and communicate information. Robots are viewed as embedded agents that can interact with humans or with other robots in a socially acceptable manner, also known as social robots [49,50]. Wearables refer to technologies that can be worn on the human body, such as virtual reality (VR) headsets, fitness trackers, smart watches, or smart jewelry. The term “smart home” (or “smart house”) refers to a residence equipped with “smart technology,” namely a variety of internet-connected sensors and systems enabling monitoring and management to automate and optimize control of the home environment, home appliances, and the inhabitant’s quality of life [51].

Previous reviews have also generally reflected the effectiveness of social robots in elderly care, including studies that address loneliness or social isolation [52,53], and the influence of smart houses on older adults’ quality of life, including their effect on social isolation [54]. Recently, in the face of the COVID-19 pandemic, there has also been a stream of studies from different fields reviewing and considering the importance and possibilities of robots and computer agents in alleviating loneliness [55-58]. To the best of our knowledge, no prior studies have focused on role of physical ICT solutions in assessing and combating loneliness and social isolation among older adults.

When the solutions relate to health and care, the concept of ambient assisted living (AAL) comes into play. AAL is a subarea of ambient intelligence and can be defined as “an emerging multidisciplinary field aimed at providing an ecosystem of different types of sensors, computers, mobile devices, wireless networks, and software applications for personal health care monitoring and telehealth systems” [59]. AAL was first coined in 2006 by the International Medical Informatics Association in recognition of this emerging technology with the creation of a working group on smart homes and AAL [60].

The aim of this study is to gain insight into how physical ICTs can help overcome loneliness and social isolation among older adults other than by fostering social communication with people and what the main open-ended challenges according to the reviewed studies are. Our focus is on empirical research conducted from January 2006 to late May 2021, starting from the year in which the concept of AAL was introduced. In line with these aims, we established the following research questions:

(RQ1) What has been studied so far, from a sociotechnological perspective, in the field of loneliness and social isolation in older adults using physical ICT solutions?

(RQ2) How can physical ICT solutions help overcome the issues of loneliness and social isolation among older adults other than by fostering social communication with people?

(RQ3) What are the main open-ended challenges according to existing studies?

## Methods

### Data Collection

A systematic literature review was conducted to answer our research questions. The PRISMA (Preferred Reporting Items for Systematic Reviews and Meta-Analyses) procedure [61] was followed, when applicable, for the study objectives. The data were collected in 2 phases. The first phase took place in April 2020, covering the period from January 2006 to late March 2020. The second phase was conducted in June 2021, covering the time frame from April 2020 to the end of May 2021, which allowed us to keep the data up to date.

In both phases, the procedure was the same. We used 7 bibliographic databases: Scopus (Elsevier), Web of Science (Clarivate), EBSCOhost (EBSCO), Social Science Premium Collection (ProQuest), PsycINFO (Ovid), PubMed (National Library of Medicine), and IEEE Xplore Digital Library (IEEE), with all databases selected. The following search phrases were used in the databases: (“ambient assisted living” OR “ambient intelligence” OR “smart house” OR “smart home” OR “smart environment” OR “smart assistant*” OR “intelligent assistant*” OR sensor* OR “internet of things” OR wearable* OR robot* OR “artificial intelligence”) AND (eld* OR age* OR old* OR geriatr* OR senior*) AND (lone* OR “social* isolat*”).

The search was targeted toward the “title,” “abstract,” and “keywords.” In PsychINFO, “key concepts” were selected as corresponding to keywords. In Social Science Premium Collection, “all subjects and indexing” including keywords and index terms was the term used in addition to abstracts and document titles. EBSCOhost and Social Science Premium Collection searches were filtered to include only peer-reviewed entries, and IEEE Xplore Digital Library was filtered for conference and journal publications to manage the number of irrelevant entries. All searches were limited to English language publications.

In the first phase, the search from the 7 different databases first produced 1830 results in total. After removing duplicate results, the data consisted of 1001 entries. In the second phase, the search resulted in 559 entries. After removing the duplicates and overlaps with data from the first phase, the additional data consisted of 270 entries. In both phases, all papers were screened according to the predefined inclusion criteria. The studies were included based on the following criteria:

(C1) It is a peer-reviewed article or a conference publication.

(C2) It is a fully empirical study, using quantitative, qualitative, or mixed methods.

(C3) The study does fully or partially research older adults.

(C4) The study does fully or partially research human loneliness or social isolation.

(C5) The study does fully or partially research physical ICTs (robots, wearables, or smart homes and houses).

Consequently, we focused on empirical studies in which physical ICT solutions are researched with older adults and loneliness or social isolation is explicitly examined. To be included, ICT solutions had to be physically exploited and studied in relation to older adults’ loneliness and social isolation, and not solely aimed at mediating or fostering communication between people. All study participants referred to as older adults in the selected studies were considered eligible for our study purposes (starting from people over 50 years old). Due to a technology-focused research topic, all relevant studies were searched, including peer-reviewed journal and book articles, as well as conference publications. Studies were excluded if they were theoretical articles or literature reviews; if they were whole books, editorials, commentaries, study protocols, or patents; if they did not explicitly mention older adults, loneliness, social isolation, or any physical ICT solution; or if the full text was not written in English.

In the first search phase, 2 coders first independently reviewed the papers’ titles, abstracts, and keywords, after which selections based on the predefined inclusion criteria were made. An interrater reliability test was conducted and resulted in an interrater agreement of 94.57% on average (mean Cohen κ=0.83, range 0.74-0.88), indicating a successful set of criteria. Borderline cases and disagreements were discussed with the members of the research team. Then, 66 full papers were screened by 2 coders against the predefined inclusion criteria, of which 17 papers were included in the data set.

**Figure 1:**
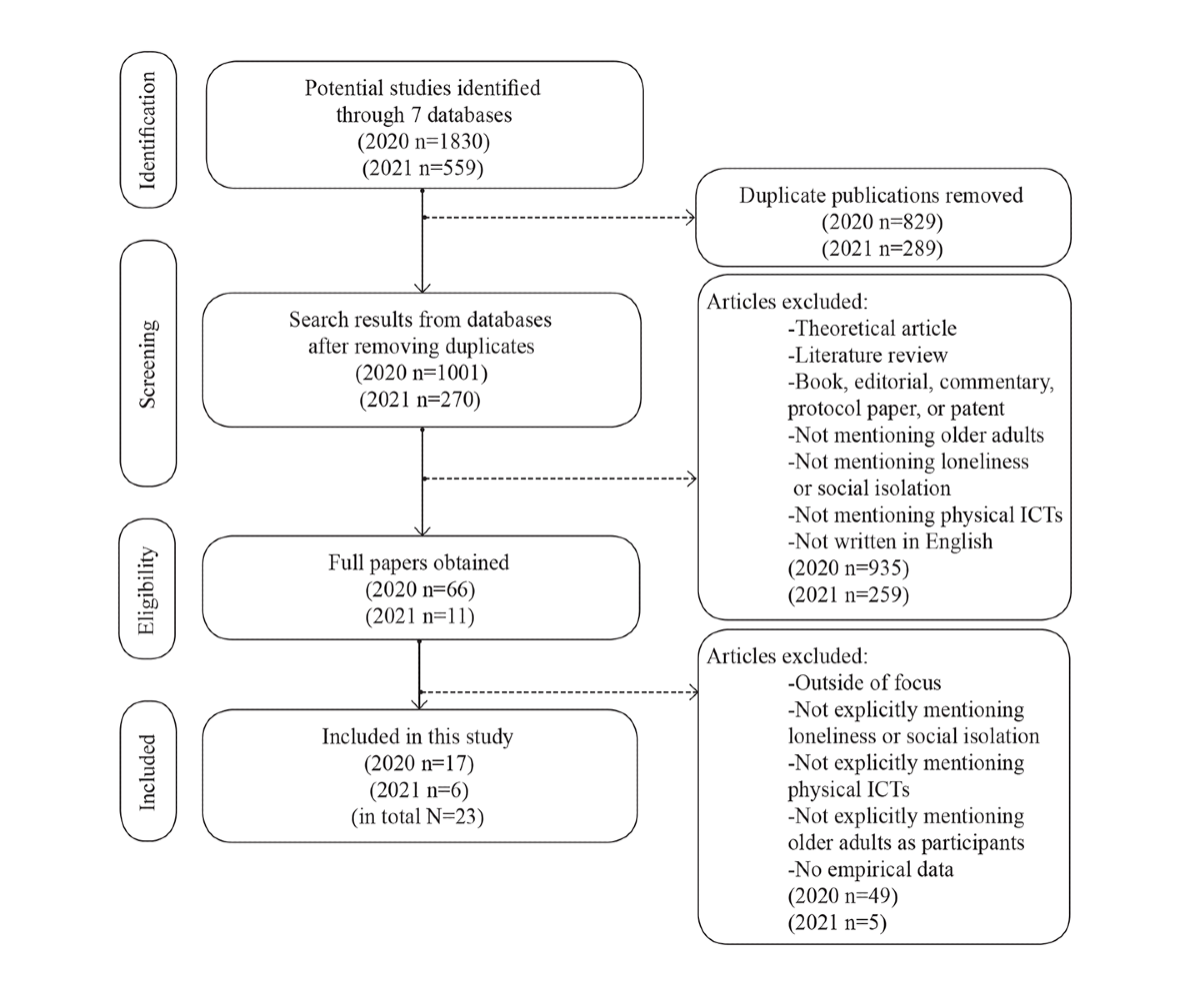
Diagram of the entire data collection and data selection process including both search phases. ICTs: information and communication technologies.

In the second search phase, we extended data collection until the end of May 2021, and the same procedure was followed. An interrater reliability test resulted in an interrater agreement of 94.67% on average (mean Cohen κ=0.79, range 0.48-1.00), replicating the success of the set criteria. Further, 11 full papers were independently screened by 2 coders, of which 6 papers were included in the data set. Hence, the final data set consisted of 23 empirical studies. The diagram depicting the entire data collection and data selection process is presented in Figure 1.

### Method of Analysis

We began the analysis by examining what has been studied from a sociotechnological perspective in the field of loneliness and social isolation in older adults using physical ICT solutions. A descriptive overview of the studies was obtained to include the following basic characteristics of the studies coded into an Excel sheet (Microsoft Corporation): research method (quantitative, qualitative, or mixed), type of study, time frame, study setting, older adults’ sample size, age, and gender information, research instrument for measuring loneliness or social isolation, type of physical ICT addressed, and focus of the paper (detection or prediction, alleviation, or other). Then, content analysis was performed to summarize the ways in which new technologies can help overcome issues of loneliness and social isolation among older adults and the main open-ended challenges according to the included studies. Meta-analysis was not conducted due to the heterogeneity of the reviewed technologies and methods used in these studies. Furthermore, our methodological choices were limited by the small number of existing studies on the topic.

## Results

### Descriptive Details of the Reviewed Studies

**Figure 2:**
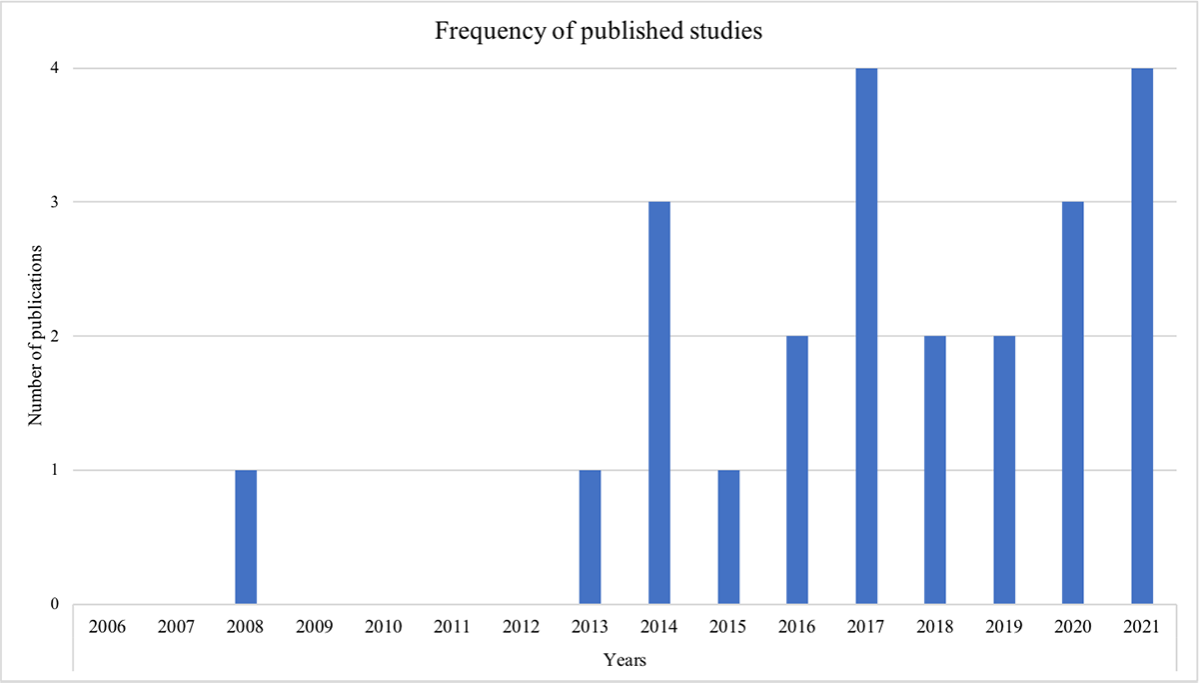
Frequency of publications per year from 2006 to the end of May 2021.

The final data set consisted of 23 studies conducted from 2006 to the end of May 2021. Except for 1 study, all the others were published after 2012. This suggests that the amount of research has increased over the past decade, as depicted in Figure 2. Studies have mostly been conducted in the United States (n=10), Germany (n=2), and Singapore (n=2). Other countries include Australia, Canada, Ireland, Mexico, the Netherlands, New Zealand, and Taiwan. Among these, 2 studies included cross-national data, 1 with participants from the United Kingdom, Italy, and Ireland, and another with participants from England and Japan. Most of the studies have applied quantitative (n=13) or mixed methods (n=7), whereas very few studies have applied qualitative methods (n=3). Most of the included studies contributed to the field of older adults’ loneliness (n=17), whereas the rest examined social isolation or both phenomena (n=6).

### Main Areas of Research

The 2 main areas of the reviewed research comprised “detection and prediction” and “alleviation” of older adults’ loneliness and social isolation using physical ICT. Here, the category of detection and prediction refers to studies that hypothesize the potential of certain activities or daily habits (attributes), including time spent in certain rooms of the house and outdoors, to infer the levels of loneliness or social isolation in older adults. In the reviewed studies, certain daily activities are tracked in real time through physical ICTs, namely smart home solutions, or a combination of smart homes and smartphones. Then, algorithms derive behavioral patterns from the gathered data. Afterward, these factual scores are correlated with subjective standard measurements to identify the attributes that meaningfully relate to loneliness or social isolation. In addition, studies validate their predictive models using different evaluation methods and indexes.

Alleviation studies include examining whether the use of technology would result in reducing perceived loneliness or social isolation in older adults, or the possible roles physical ICT could play in combating older adults’ loneliness and social isolation, if not serving as an actual intervention. Further, 1 paper was categorized as “other,” referring to a study examining the association between perceived loneliness and acceptance of robots. It was also included in the final data set, as it has implications in terms of overcoming loneliness from an opposite perspective; without acceptance of the intervention for reducing perceived loneliness, there is no possible cure.

The focus was on detection and prediction in less than half of the studies (n=7), whereas the alleviation of loneliness or social isolation was the focus in most of the considered studies (n=15). None of the studies directly examined both aspects. The basic information of the included studies is presented in Table 1.

**Table 1:** Basic information of the selected studies (N=23).

Method and context		Focus			
		Detection, n (%)	Alleviation, n (%)	Other, n (%)	Total, N (%)
**Method**					
	Quantitative	6 (85.71)	6 (40)	1 (100)	13 (56.52)
	Qualitative	—^a^	3 (20)	—	3 (13.04)
	Mixed method	1 (14.29)	6 (40)	—	7 (30.44)
	Total	7 (100)	15 (100)	1 (100)	23 (100)
**Study context and countries**					
	United States	3 (42.86)	7 (46.67)	—	10 (43.48)
	Singapore	2 (28.57)	—	—	2 (8.7)
	Germany	—	1 (6.67)	1 (100)	2 (8.7)
	Australia	—	1 (6.67)	—	1 (4.35)
	Canada	—	1 (6.67)	—	1 (4.35)
	Ireland	1 (14.29)	—	—	1 (4.35)
	Mexico	1 (14.29)	—	—	1 (4.35)
	The Netherlands	—	1 (6.67)	—	1 (4.35)
	New Zealand	—	1 (6.67)	—	1 (4.35)
	Taiwan	—	1 (6.67)	—	1 (4.35)
	United Kingdom, Italy, and Ireland	—	1 (6.67)	—	1 (4.35)
	England and Japan	—	1 (6.67)	—	1 (4.35)
	Total	7 (100)	15 (100)	1 (100)	23 (100)

^a^—:not available

### Types of Technologies Assessed

Within the reviewed studies on the detection and prediction of loneliness and social isolation, platforms with several types of sensor-based technologies were commonly used, namely, smart home solutions. Austin et al [62], Goonawardene et al [63], Petersen et al [64,65], and Walsh et al [66] assessed smart home solutions explicitly, whereas Huynh et al [67] analyzed sensor-enabled homes. Martinez et al [68] combined smart homes and smartphones in their study. In the reviewed studies focusing on loneliness and social isolation alleviation, older adults have been introduced most often to social robots [69-79]. Studies have also examined the use of a smart home solution [80], VR systems [81,82], and an ambient activity system that includes an activity sensor (AAL-VU system) [83]. Furthermore, 1 study focusing on how loneliness associates with physical ICT acceptance exploited social robots Paro and Giraff [84].

### Study Designs and Settings

The reviewed studies applied various study designs ranging from explorative pilot studies to randomized controlled experiments. Longitudinal design was applied in some studies [62,65], and 3 studies followed the randomized controlled trial protocol [69,76,77]. A majority of the 23 studies applying quantitative measurements used validated scales for subjectively measuring loneliness and social isolation: the original, revised, or short version of the University of California Los Angeles (UCLA) Loneliness Scale (n=12), Dong Jong Gierveld Loneliness Scale (n=4), or Lubben Social Network Scale (n=2). However, most studies were conducted with relatively small sample sizes, which is also understandable due to the use of new technologies and the experimental nature of the studies. Many studies targeted healthy older adults with no cognitive impairment with the exceptions of Appel et al [81], focusing on older adults with cognitive and physical impairments; Robinson et al [77], in which 19 out of the 40 participants had cognitive impairment; Fields et al [72], where roughly half of the participants had dementia; and Casey et al [70] focusing on people with dementia. Further, Chen et al [71] focused on older adults with depression, and Hudson et al [74] and Tkatch et al [79] noted that their participants had higher levels of depression compared to the ones who declined from participating in the study. Studies were conducted in the older adults’ own homes, different forms of care facilities, sensor-enabled houses and apartments, and hospitals. Table 2 gives a descriptive overview of the considered studies.

**Table 2:** Descriptive overview of the selected studies (N=23).

Studies and method used	Type of study	Time	Setting	Environment	No. of participants	Age in years	Female (%)	Instrument	Type of technology and focus
Appel et al [81]; mixed	Feasibility	3×20 min^a^	Facility	Indoors	66	Mean 80.5	60. 6	1 item in STAI^b^	VR^c^ system; alleviation
Austin et al [62]; quantitative	Longitudinal	8 mo^d^	Home	Indoors	16	>62 (mean 71)	81	UCLA^e^	Smart home; detection
Baisch et al [84]; quantitative	User/field study	N/A^f^	N/A	Indoors	29	65-81 (median 70)	79	1 item	Paro and Giraff robots; other
Banks et al [69]; quantitative	RCT^g^	8 wk^h^	Facility	Indoors	38	N/A	N/A	UCLA	AIBO robot; alleviation
Brandenburgh et al [83]; mixed	Feasibility	6 wk	Home	Indoors	Multip.^i^	Multip.	Multip.	DJGLS^j^	AAL-VU^k^ system; alleviation
Casey et al [70]; qualitative	Interview	2 mo	Home, facility, hospital	Indoors	38	Multip.	Multip.	Interview	MARIO robot; alleviation
Chen et al [71]; mixed	Quasi-experiment and interview	8 wk	Facility	Indoors	20	65-93 (mean 81.1)	65	UCLA	Paro robot; alleviation
Curumsing et al [80]; mixed	Case study, field trial	13 wk	Home	Indoors	10	>65	N/A	Interview	Smart home; alleviation
Fields et al [72]; quantitative	Pilot experiment	3×10 min	Facility	Indoors	15	77-92 (mean 85.80)	73.3	UCLA	NAO robot; alleviation
Follman et al [73]; mixed	Experiment and interview	2 mo	Facility, hospital	Indoors	70	59-98 (av.^l^ 83)	72.86	UCLA	temi robot; alleviation
Goonawardene et al [63]; mixed	Multimethod	7 mo	Smart home	Indoor/outdoor	46	60-91	58.7	LSNS^m^, DJGLS, social activity attendance	Smart home; detection
Hudson et al [74]; qualitative	Interview	1 h^n^	Home	Indoors	20	65-90 (av. 76)	50	Interview	Pet robots; alleviation
Huynh et al [67]; quantitative	Field study	6 mo	Smart home	Indoor/outdoor	43	Mean 77.59	N/A	DJGLS	Smart home; detection
Lazar et al [75]; qualitative	Focus group	N/A	Home, facility	Indoors	41	61-92 (mean 77)	85.37	Interview	Pet robots; alleviation
Lin et al [82]; quantitative	Field study	2 wk	Facility	Indoors	63	Born 1918-1950	62	UCLA	VR system; alleviation
Martinez et al [68]; quantitative	Multimethod	N/A	Home	Indoors/outdoors	Multip.	Multip.	Multip.	LSNS	Smart home; smartphone; detection
Papadopoulos et al [76]; quantitative	RCT	18 h across 2 wk	Facility	Indoors	33	65-98 (mean 81.9)	66.7	UCLA (ULS-8^o^)	Pepper robot; alleviation
Petersen et al [64]; quantitative	Multimethod	5 d^p^	Facility, home	Outdoors	Multip.	Multip.	Multip.	UCLA	Smart home; detection
Petersen et al [65]; quantitative	Longitudinal	12 mo	Facility, home	Outdoors	85	65-96 (mean 86.36)	85	UCLA	Smart home; detection
Robinson et al [77]; quantitative	RCT	12 wk	Facility	Indoors	40	55-100	N/A	UCLA	Paro robot; alleviation
Sidner et al [78]; mixed	Field study	1 mo	Home	Indoors	44	55-91 (mean 66)	N/A	UCLA	AlwaysOn, robot/virtual; alleviation
Tkatch et al [79]; quantitative	Intervention	1 mo	Home	Indoors	216	65-85	Multip.	UCLA	Animatronic/robotic pets; alleviation
Walsh et al [66]; quantitative	Field study	4×28 d	Smart home	Indoors	13	60-88	46.15	DJGLS	Smart home; detection

^a^min.: minutes.

^b^STAI: State-Trait Anxiety Inventory.

^c^VR: virtual reality.

^d^mo: month or months.

^e^UCLA: University of California Los Angeles Loneliness Scale.

^f^N/A: not applicable/not mentioned.

^g^RCT: randomized controlled trial.

^h^wk: week or weeks.

^i^Multip.: multiple data.

^j^DJGLS: Dong Jong Gierveld Loneliness Scale.

^k^AAL-VU: an ambient system.

^l^av.: average.

^m^LSNS: Lubben Social Network Scale.

^n^h: hour or hours.

^o^ULS-8: UCLA Loneliness Scale-8.

^p^d: day or days.

### Detecting and Predicting Older Adults’ Loneliness and Social Isolation via Behavioral Attributes

The studies aiming to overcome loneliness and social isolation through detection and prediction are framed within the assumption that an early diagnosis of loneliness and social isolation in older adults can prevent their physical and psychosociological decline. All 7 papers report relevant results and provide a meaningful quantitative or qualitative evaluation of their experiments. All the solutions are shown, at least to some extent, to be capable of detecting and predicting older adults’ loneliness or social isolation; thus, they help in the process of overcoming such circumstances by recognizing the existence of these phenomena. Overall, researchers refer to these detection systems as promising research paths toward overcoming loneliness and social isolation.

In practice, studies assess various older adults’ behavioral attributes and compare them with subjective measures. Older adults’ out-of-home habits measured in 5 of the studies [63-65,67,68] are reported as relevant attributes in inferring loneliness and social isolation. Thus, tracking attributes related to outings (time spent outside the house, number of outings, and number of places visited) seems consistently relevant in unobtrusive models to detect loneliness or social isolation. Nevertheless, other attributes analyzed in these studies are not as consistent across the studies in terms of their relevance to detection. Different studies report that diverse variables should be considered in prediction models with this type of technology. Time spent in the house is generally relevant in 1 study [68], whereas it is not in another [62]. Other reported significant variables include time spent in the living room [63,66], time spent across various locations [66], walking speed [62], nocturnal movements [66], and daytime napping [63].

Studies vary in terms of the development phase of the system. Martinez et al [68] propose a smartphone app that enables the users and caregivers to interact and receive feedback from the system, whereas Huynh et al [67] and Walsh et al [66] elaborate on potential interfaces. Some studies expand on the core issue of loneliness and social isolation inference. Huynh et al [67] also assess depression among independently living seniors in their study. Petersen et al [65] focused on monitoring the broad cognitive, physical, and emotional states of older persons, and Walsh et al [66] also explored anxiety, cognition, depression, independent living skills, sleep quality, and quality of life.

Papers also report some study-related issues, such as small sample sizes [62,66,68], study durations [65,67], and nonoptimal study designs [62,65]. Likewise, some studies report technical problems, such as ensuring that sensor configurations work continuously [66], assessing situations with more than one person living in a house [66,67], and the intrusiveness of smartphone models [68]. Moreover, 2 studies note general ethical challenges regarding issues such as privacy, respect, or consent [62,66].

### Alleviating Older Adults’ Loneliness and Social Isolation Through Physical ICT

Studies on alleviation indicate that some physical ICT solutions can help overcome loneliness among older adults by decreasing it based on self-reported measures. None of the studies report complete elimination of older adults’ loneliness experiences with the help of physical ICT, but they report success in alleviating them. Studies that report statistically significant results for a decrease in perceived loneliness of older adults include interventions using social robots [69,71-73,77,79] and an ambient activity system including an activity sensor [83]. Moreover, 2 studies report qualitative evidence of using social robots in alleviating loneliness [70,74]; in addition, 1 study reports unexpected qualitative evidence regarding the unexpected positive impacts of an intervention using a smart home solution on loneliness [80]. No statistically significant results were demonstrated for a decrease in social isolation, but 2 studies suggest that social robots could help combat social isolation by providing social contact with the robots and through video calls with people [70,73].

Due to the variety of physical ICTs employed in the studies on loneliness alleviation, common characteristics regarding intervention designs or technological features are limited. However, a common feature of the successful solutions is that they can interact with the user in one way or another. The successfully employed social robots (Paro, AIBO, NAO, MARIO, temi, and animatronic or robotic pets) are all designed to engage with the user, thus providing means for interaction with the device itself. MARIO and temi robots also provide means for video communication with other people as a central feature besides speech, activity, and entertainment capabilities. In an ambient activity stimulating system [83], a virtual coach gives recommendations to the user based on the planned activities for the day and the data from an activity sensor. The smart home solution [80] also interacts with the user via messages.

Banks et al [69] assessed the mechanism leading to changes in loneliness and report that the intervention did not succeed in alleviating loneliness through attachment to the robotic dog (AIBO) or a living dog. Hudson et al [74] report that many of their participants stated that the presence of the robotic pet positively influenced their feelings of loneliness. Curumsing et al [80] also report that older adults missed the voice of that “somebody” in their home when they uninstalled the smart house system. Casey et al [70] describe that their participants thought that MARIO reduced their feelings of loneliness because the robot provided distractions, allowed them to engage in various activities and interact with family members.

However, the evidence found by these studies must be interpreted cautiously. Studies acknowledge that more valid results would have been obtained with larger sample sizes [70,72,73,76,77], and the lack of control or comparison groups limits the interpretations of the effectiveness of the intervention [71,72,77,79]. Scholars also recognize the possible influence of the presence of a researcher on their results [70,72,76,77] and raise concerns on study dropouts due to older adults’ health issues [77]. Robinson et al [77] performed the only study conducting a group session, and they reflect on the possible effects of that group setting on the intervention in a subsequent study [85]. Banks et al [69] noticed that the robotic dog AIBO was not used at full capacity in the study, which may have influenced the results. Further, 6 studies using social robots included participants with cognitive impairment [77], dementia [70,72], and depression [71,74,79], which must also be acknowledged when interpreting the results. Curumsing et al [80] did not focus on alleviating loneliness in their initial study plan, but the topic emerged during the investigation, leading to unexpected results.

### Perceived Loneliness in Technology Acceptance

One study shed light on the link between perceived loneliness and acceptance of robots. Baisch et al [84] investigated emotional loneliness as a component of psychosocial functioning and its link with the intention to use social robots Paro and Giraff in 2 different user-technology fit scenarios. According to the results, participants with lower psychological resources, including perceived emotional loneliness, accepted the Giraff robot less when the user-technology fit was poor. The same results were not obtained for a companion robot. However, researchers warn against generalizing the results due to a small and nonrepresentative sample.

### Main Open-Ended Challenges According to the Reviewed Studies

The main common open-ended challenges according to the reviewed studies relate to the need for more robust study samples and study designs. Across studies, researchers suggest future studies using larger study samples that could also be more diverse [62,65] and more geographically widespread [77,82], include more people with different conditions [77], and vary more in terms of the living environments [77,82] to add statistical power for generalization, to validate the interpretation of results, and to understand for whom the technologies are best suited. However, researchers admit that realistically obtaining larger samples includes challenges such as the health problems of the seniors [77] and finding isolated adults for recruitment in general [66,78]. Further, it is critical for future studies to incorporate control and comparison groups and confounding factors to the study designs to aid the interpretation of the effectiveness of the interventions [71,72,76,77,79]. Finally, scholars note that research would benefit from longer study periods [63,65,67,70,73,74,76,84].

Among the detection and prediction studies, Goonawardene et al [63] and Petersen et al [64] propose introducing potentially confounding variables (eg, depression or mobility) and establishing causal relationships between them, applying multivariate estimation models to improve accuracy. For accuracy purposes, Walsh et al [66] recommend tracking the exact day when data are gathered instead of relying on average measurements, and Petersen et al [65] encourage implementing detailed environmental variables (eg, weather conditions, seasonal changes, proximity of resources, ease of transportation, living alone or within a community, or neighborhood demographics).

Regarding the alleviation interventions, more knowledge is needed regarding the effect of group sessions on the intervention results [77,85] to ascertain whether to conduct studies individually or in group settings [72]. The need for advanced and low-cost robotic devices to be used for research purposes [72,75] sets an open challenge for the growing robot market. The study by Lazar et al [75] challenges the perception of seeing pet robots as “technological fixes” for loneliness or social isolation alleviation; instead, it suggests reimagining their potential to suit specific needs and existing social lives. Brandenburgh et al [83] state that future developments may focus on how to change human behavior in addition to which behavior should be changed.

Regarding technological features, Appel et al [81] mention open challenges for future VR studies to succeed better in alleviating feelings of being lonely by applying joint or multiuser experiences with multidisplay setups. Lin et al [82] state that other VR-related systems and technologies could also be examined, but they do not specify the types of systems. Casey et al [70] suggest that robots used in dementia care could have more human-like features and better capabilities for communication and understanding speech in future. Chen et al [71] advocate a reliable method for measuring interaction time, such as an in-built function in Paro. Tkatch et al [79] propose effect comparisons between having a real pet or a robotic pet, and between a robotic cat and a robotic dog. Hudson et al [74] suggest implementing robotic pets with increased interactivity in future interventions; however, they also raise a potential ethical issue where older adults become dependent on their pet. Sidner et al [78] add that instead of using predetermined methods for introducing activities in the system, more flexible and teachable methods could be sought to prevent failures during activity usage in future.

Baisch et al [84] recommend more comprehensive research on the psychological mechanisms and human characteristics with respect to the acceptance of robots, as well as the diversity of older adults’ life circumstances. The stage is also open for new ideas to enhance the perceptions of user-technology fit among older adults.

## Discussion

### Principal Results

This systematic literature review examined the research conducted on older adults’ loneliness and social isolation and physical ICT solutions in the era of AAL. The aim was to gain insight into how technologies can help overcome such circumstances without fostering social communication with people and what the main open-ended challenges according to the included studies are. The results demonstrate that issues of loneliness and social isolation among older adults cannot be eliminated using physical ICTs, but that physical ICTs are used to help detect and predict, or alleviate such circumstances. ICT solutions such as smart homes can help predict and detect loneliness and social isolation, and technologies such as robotic pets and some other social robots can help alleviate loneliness to some extent. The main common open-ended challenges according to the reviewed studies are related to the need for more robust study samples and study designs. In addition, studies reported some technology- and topic-specific open-ended challenges.

Based on our findings, all the reviewed smart home and house solutions in the area of detection and prediction were considered capable of detecting and predicting older adults’ loneliness or social isolation to some extent. Tracking the outings, including the time spent outside the house, number of outings, and number of places visited, stood out as a relevant activity to be examined in the unobtrusive models. Overall, these methods show a promising research path for overcoming loneliness and social isolation.

Open-ended challenges in the area of detection and prediction included a more flexible adaptation of predictive models to contingencies and contextual situations and the development of learning algorithms that allow the systems to accurately respond to the evolving circumstances of older adults. In addition, ethical issues posed by the intrusiveness of monitoring systems in older adults’ lives, as well as economic concerns, remain to be assessed. Further, as pointed out in one of the studies [65], it is relevant that researchers continue to find a way to implement the complexity and variety of living spaces in their experiments if they seek accuracy in their results and solutions to overcome loneliness and social isolation. It is clear that the environment shapes individuals, and thus, these technologies could be exploited further to “attune” [86] the built environment with “typical human situations” [87] to contribute toward increasing the social inclusion of older adults and alleviate feelings of loneliness.

According to the results, some forms of physical ICT hold promising value in alleviating older adults’ loneliness. Significant positive results were obtained in interventions using social robots, an ambient activity system that includes an activity sensor, and a smart home solution. A common feature of these solutions is that they are able to interact with the user in one way or another, which may have served as the key to their success by fostering reciprocity between the device and the user, thus providing increased social contact opportunities for older adults. Some studies suggest that social robots could also help reduce social isolation by providing social contact and activities for older adults, but more robust evidence is still needed to prove that social contact is being increased and not being replaced by technology. One reviewed study demonstrated that the experience of loneliness itself as part of psychological functioning was associated with lower acceptance of a telepresence robot in a poor user-technology fit scenario. Future studies should continue investigating the role of subjective feelings of loneliness in technology acceptance and adoption longitudinally to draw comprehensive conclusions.

Open-ended challenges related to alleviation included uncertainty about whether the interventions should be conducted individually or in group settings and the high cost of technologies. Regarding technological features, such as VR, joint or multiuser experiences with multidisplay setups could be explored in future. For social robots, scholars seek enhanced interaction and speech recognition capabilities, possibly in-built means for measuring interaction time, and further comparisons on the effectiveness of different robotic pets. There is also a need for more flexible and teachable methods for systems aiming to alleviate loneliness and developing knowledge on how technologies can be used for changing human behavior.

Furthermore, we found that none of the solutions in the reviewed studies aimed to alleviate and detect as well as predict older adults’ social isolation or loneliness explicitly. Some of the solutions are perhaps able to do so, but none of the studies have examined both aspects by empirical methods. Solutions to assess and combat social isolation have so far been less researched compared to loneliness and are particularly needed. The need is evident in the COVID-19 era with on-going lockdowns and social restrictions all over the world, and where fast digitalization and ICT adoption on large scales have also happened at an unprecedented pace [88]. As discussed by Christina R Victor [89], scholars may also shift from a problem-based focus to preventive aspects, namely toward enhancing healthy social habits. Along these lines, the theoretical framework of the studies might be enriched, going beyond this consideration of loneliness as a contingent and transient or chronic experience by embracing other philosophical perspectives that consider it to be the existential condition of each of us; thus, loneliness is a permanent and unavoidable condition generated by the activity of consciousness [90].

### Limitations

This study explicitly concentrated on loneliness and social isolation among older adults. Thus, it contributes most in situations where loneliness and social isolation can be suspected or are already present among older adults, which may be seen as a limitation of this study. In addition, the existing setup could be broadened to include other dimensions related to loneliness and social isolation, and other technologies to yield larger data sets for future reviews. For instance, there are studies using smartphones for detecting and predicting older adults’ loneliness and social isolation [91,92]; studies also consider numerous forms of technical systems different from the ones we considered, aiming to reduce older adults’ loneliness [93,94]. There are also other studies conducted regarding the effects of loneliness on technology adoption [95], and those neither exploiting physical ICTs [96,97] nor assessing the technology directly in relation to loneliness or social isolation but in terms of related factors [98,99]; these are beyond the scope of this review but closely related to it. However, we did not include all possible technologies and approaches in our study to keep it focused.

### Comparison With Prior Work

This review differs from other reviews addressing ICT technologies to assess older adults’ loneliness and social isolation focusing on mediating human-human interactions. It addresses ICTs that perform by themselves and do not aim to foster human relationships as the main objective of their interventions. This study also complements some other systematic literature reviews by addressing partially overlapping technologies, namely robots and smart houses; thus, it provides a more comprehensive view of the research in this regard. Further, to our knowledge, this review is the first one compiling the studies on detection and prediction of older adults’ loneliness and social isolation, which is an emerging field that has received only little attention thus far [100].

### Conclusions

This paper provides a comprehensive overview of the existing attempts aiming to combat older adults’ loneliness and social isolation using physical ICTs, namely robots, wearables, and smart homes. Our findings demonstrate that physical ICTs such as smart home solutions can help detect and predict loneliness and social isolation, and technologies such as robotic pets and some other social robots can help alleviate loneliness to some extent. Technologies such as social robots and some of the smart home solutions that can react to human behavior or interact with people are promising, and the literature on these topics has increased recently. These findings have relevant implications to the discussion on combating loneliness and social isolation among older adults that challenge the prejudices about the use of technology in this sensitive area. The results benefit the academic community by accumulating research evidence and finding future research targets, as well as informing other professionals and practitioners of the current state of research, developed solutions, and interventions conducted. The results are also useful in the COVID-19 era, where it is extremely important to find solutions to cope with social isolation and loneliness.

## Abbreviations

AAL: ambient assisted living
ICT: information and communication technology
UCLA: University of California Los Angeles
VR: virtual reality
